# Age exacerbates microglial activation, oxidative stress, inflammatory and NOX2 gene expression, and delays functional recovery in a middle-aged rodent model of spinal cord injury

**DOI:** 10.1186/s12974-017-0933-3

**Published:** 2017-08-18

**Authors:** Ramona E. von Leden, Guzal Khayrullina, Kasey E. Moritz, Kimberly R. Byrnes

**Affiliations:** 1 Neuroscience Program, Uniformed Services University, 4301 Jones Bridge Road, Bethesda, MD 20814 USA; 2 Department of Anatomy, Physiology, and Genetics, Uniformed Services University, Room C2099, 4301 Jones Bridge Road, Bethesda, MD 20814 USA

**Keywords:** Spinal cord injury, Aging, NOX2, Microglia, Inflammation

## Abstract

**Background:**

Spinal cord injury (SCI) among people over age 40 has been steadily increasing since the 1980s and is associated with worsened outcome than injuries in young people. Age-related increases in reactive oxygen species (ROS) are suggested to lead to chronic inflammation. The NADPH oxidase 2 (NOX2) enzyme is expressed by microglia and is a primary source of ROS. This study aimed to determine the effect of age on inflammation, oxidative damage, NOX2 gene expression, and functional performance with and without SCI in young adult (3 months) and middle-aged (12 months) male rats.

**Methods:**

Young adult and middle-aged rats were assessed in two groups—naïve and moderate contusion SCI. Functional recovery was determined by weekly assessment with the Basso, Beattie, and Breshnahan general motor score (analyzed two-way ANOVA) and footprint analysis (analyzed by Chi-square analysis). Tissue was analyzed for markers of oxidative damage (8-OHdG, Oxyblot, and 3-NT), microglial-related inflammation (Iba1), NOX2 component (p47^PHOX^, p22^PHOX^, and gp91^PHOX^), and inflammatory (CD86, CD206, TNFα, and NFκB) gene expression (all analyzed by unpaired Student’s *t* test).

**Results:**

In both naïve and injured aged rats, compared to young rats, tissue analysis revealed significant increases in 8-OHdG and Iba1, as well as inflammatory and NOX2 component gene expression. Further, injured aged rats showed greater lesion volume rostral and caudal to the injury epicenter. Finally, injured aged rats showed significantly reduced Basso–Beattie–Bresnahan (BBB) scores and stride length after SCI.

**Conclusions:**

These results show that middle-aged rats demonstrate increased microglial activation, oxidative stress, and inflammatory gene expression, which may be related to elevated NOX2 expression, and contribute to worsened functional outcome following injury. These findings are essential to elucidating the mechanisms of age-related differences in response to SCI and developing age-appropriate therapeutics.

## Background

There are over 250,000 people in the USA currently living with a spinal cord injury (SCI), and over 15,000 new cases are documented every year [1]. These injuries cause permanent motor, autonomic, and sensory function loss [2]. Reduced cell viability and neuron, astrocyte, and oligodendrocyte loss are seen acutely after SCI [3]. White matter and axon loss, glial scar formation, and inflammation are seen chronically after injury, in the days to months following onset [3].

SCI is largely studied in the young adult population due to the high propensity of injuries from athletic and military injuries and car accidents; however, there has been a notable increase in age of onset in recent decades [4]. In the 1970s, the average age at onset of injury was 29, but the average age has now increased to 42 [1]. Aging tissue shows progressive DNA damage beginning by the age of 40, causing increased expression of genes involved in stress response and repair, including immune and inflammatory genes [5]. Further, aging causes changes to hormones and cellular function associated with increased co-morbidities, lengthened recovery time, reduced functional recovery, and prolonged chronic inflammation after SCI [6].

One notable change to the cellular environment with aging is an increase in oxidative stress caused by the buildup of reactive oxygen species (ROS) over time [7, 8]. The superoxide producing NADPH oxidase (NOX) enzyme is a major source of ROS [9]. The NOX2 isoform has been shown to be the most responsive to injury and is present on microglia, the primary inflammatory cell type of the central nervous system [10]. Microglia are found in a resting state in uninjured tissue, with ramified cell body and long processes. When activated, they take on a pro-inflammatory state with an amoeboid-like cell shape, releasing cytotoxic and inflammatory mediators [11]. Activated microglia are found in the spinal cord cellular environment within 24 h post-injury and have a maintained, low-level activated presence at 60 days post-injury (dpi) [11].

In animal models of traumatic brain injury (TBI), aged microglia show alterations to morphology and activation compared to younger microglia [12–14]. In uninjured aged brain tissue, microglia demonstrate a hypertrophic or partially activated cell shape, considered a “primed” activation state [14]. When injured, these aged cells show a hyperactive response to injury compared to younger cells, resulting in extended chronic inflammation [14]. Previous work has demonstrated that in the spinal cord, compared to the brain, there is a significantly greater inflammatory response and recruitment of macrophages and neutrophils to trauma [15–17]. Therefore, findings in the aging brain may not accurately indicate what will occur in the spinal cord.

Few studies have examined how age influences microglial activation and/or oxidative stress in the spinal cord [18–21] and the role NOX2 plays in this process [22]. Work in our lab has characterized the role of NOX2 in SCI [23], demonstrating that inhibition of NOX2 improves functional recovery and decreases oxidative stress and inflammation in a young adult rodent model of SCI [24]. Previous work on age-related cellular alterations after spinal cord injury have been performed in mice [22] and aged female rats [19], while in this paper we examine a middle-aged male rat model of SCI as the average age of onset of human SCI is 42 years old. Based on previous work showing age-related alterations after injury in the brain [12, 14, 25] and spinal cord [18, 19, 21, 26], we investigated microglia, NOX2 components, and ROS prior to and chronically after injury (30 dpi). We now show that microglial and NOX2 gene expression and ROS production are increased in middle-aged rats, demonstrating age-related alterations in the spinal cord.

## Methods

### Animals

Male Sprague Dawley rats were given free access to food and water and a 12-h light/12-h dark cycle. Young adult rats (3 months old: 300–500 g; Taconic, Germantown, MD: *n* = 24; Harlan, Frederick, MD: *n* = 6) from Harlan were used in addition to rats from Taconic in pilot naïve and 30 dpi injured study groups for immunohistochemistry and functional assessment. No significant difference was noted between groups from different vendors; therefore, data from all vendors are presented together. Rats were housed for 1 week prior to undergoing surgery or assessment for naïve studies. Middle-aged rats (12-months-old, retired breeders: 400–600 g; Harlan, Frederick, MD: *n* = 28) were acquired at 10 months of age and gentled twice a week until they had matured to 12 months of age, at which point they underwent surgery or assessment for naïve studies. One 12-month-old injured rat naturally perished at 20 dpi and was removed from behavioral testing data (final *n* = 17 behavioral analysis). Two 3-month-old injured rats were not included in tissue analysis as tissue was damaged in processing (final *n* = 14 tissue analysis). All 12-month-old injured rats were subjected to full behavioral battery, but the behavioral battery for the first cohort of 3-month-old injured rats did not include footprint analysis testing, which means that the total number of animals tested in the 3-month-old group was smaller than that in the 12-month-old rats (*n* = 5/10 3-month-old rats able to perform 28 dpi footprint analysis, *n* = 7/18 12-month-old rats able to perform 28 dpi footprint analysis). After functional testing, groups were divided among tissue analysis groups for histological, immunohistochemical, or biochemical analysis as designated prior to injury. All animal procedures were approved by the Uniformed Services University IACUC and complied fully with the principles set forth in the “Guide for the Care and Use of Laboratory Animals” prepared by the Committee on Care and Use of Laboratory Animals of the Institute of Laboratory Resources, National Research Council (DHEW pub. No. (NIH) 85-23, 2985); this manuscript has been written in accordance with the ARRIVE (Animal Research: Reporting In Vivo Experiments) guidelines [27].

### Contusion spinal cord injury

Contusion SCI was performed in rats (*n* = 35) (see Table 1 for breakdown of animal groups) as described previously [28]. Briefly, rats were anesthetized with isoflurane (4% induction, 2.5% maintenance in 50% O_2_), and a laminectomy was performed at vertebral level T9 to expose the spinal cord. Moderate injury (150 kDynes force, 1500-μ displacement, 127-mm/s velocity, 1-s dwell time) was induced with the Infinite Horizons Impactor (Precision Systems Incorporated, Natick, MA). Naïve animals (*n* = 22) received no surgery or anesthesia. Post-injury care included dual housing on diamond soft bedding (Harlan Laboratories, Frederick, MD), twice daily bladder expressions until spontaneous micturition returned, and analgesic supplementation to drinking water for 48 h following injury (Children’s Tylenol; acetaminophen, 200 mg/kg).

**Table 1:** Number of animals per group for all experiments in study

Number of animals per group and experiment					
Age	Injured/naïve	Function	Immunohistochemistry	Lesion volume	Biochemistry/PCR
3 months old	Naïve	12	4	0	4
12 months old	Naïve	10	4	0	4
3 months old	Injured	18	4	6	4
12 months old	Injured	17	4	6	4

Animals were first divided by age (3 or 12-months old) and injury type (naïve or injured). Functional analysis was performed on all animals in study. Animals used in function experiments were sacrificed and divided into subgroups for tissue experiments (immunohistochemistry, lesion volume, and biochemistry/PCR)

### Functional assessment

The Basso–Beattie–Bresnahan (BBB) scale was used to assess functional neurological recovery, as detailed previously [29]. Performance of the left and right hind limbs on a scale from 0 (no spontaneous movement) to 21(normal locomotion) was averaged to obtain the total BBB on 1, 7, 14, 21, and 28 dpi. More detailed locomotion was assessed using the “footprint analysis test,” modified from Kunkel-Bagden et al. [30] and performed on the same days as BBB assessment. Subjects’ hind paws were pressed to an inkpad to dye their hind feet and were then allowed to walk across a sheet of paper lining a long and narrow runway (6.5 in. wide, 76 in. long) to ensure animals walked in a straight line for analysis. Prints were analyzed for width of toe spread and length of stride length (in mm). Subjects’ footprints were only analyzed if they had regained ability to walk with support on the hind limbs (a minimum BBB score of 11), and percentage of animals who had regained the ability to perform the task was analyzed by age group at each assessment day.

### Histology and lesion volume

At 30 dpi, rats were anesthetized with Euthasol (pentobarbital sodium and phenytoin sodium; 200 mg/kg, IP) and intracardially perfused with 10% buffered formalin (Fisher Scientific, Hampton, New Hampshire). A 1-cm block of spinal cord tissue was dissected, extending 5 mm rostral and caudal from the center of the lesion site (or from the T9 vertebral site for naïve animals). Tissue was sectioned into 20-μm axial sections, and every third slice was saved for histological analyses. To determine lesion volume, tissue was processed with a standard H&E stain. Lesion volume from H&E images collected with NanoBrightfield Software (Hamamatsu Corporation, Middlesex, NJ) were quantified using the Cavalieri method as previously described [31, 32]. Ten sections were analyzed per animal at even intervals throughout the spinal cord from 360-μm pre-lesion epicenter (0–360 μm on histogram in Fig. 5a) through lesion epicenter (between 360 and 540 μm on histogram) to 360 μm post-epicenter (540–900 μm on histogram) to provide an analysis of volume of lesion throughout the spinal cord.

### Immunohistochemistry

Standard fluorescent immunohistochemistry was performed on tissue sections from animals 30 dpi obtained as described above. Iba1 (microglia, 1:100; Wako Bioproducts, Cat# 019-19741 RRID:AB_839504; Richmond, VA) and 8-OHdG (DNA oxidation, 1:500; Abcam, Cat# ab62623 RRID:AB_940049; Cambridge, MA) were used as primary antibodies, with visualization by fluorescent secondary antibodies (Alexa-Fluor Secondaries, 1:500; Molecular Probes, Eugene, OR). Negative controls (sections not stained with primary antibody) were used to confirm specificity of secondary antibodies. Immunofluorescent images were captured under the same fluorescence conditions and exposure time on a NanoZoomer system (Hamamatsu, Bridgewater, NJ) or an Olympus BX43 microscope (Olympus America). Immunofluorescence was quantified using pixel density measurement in Scion Image as previously described [33]. Ten axial sections were analyzed per animal starting caudally at the first sign of lesion, then at even intervals rostrally through the spinal cord for 1 cm to provide analysis throughout the lesion. Analysis was performed on gray matter directly adjacent to the lesion site at 20× magnification. The average pixel density of all ten sections was reported for each animal.

### Comparative RT-PCR

A 10-mm section of the spinal cord, centered at the lesion epicenter, was dissected and immediately frozen on dry ice. The TRIzol® protein and mRNA extraction method (Invitrogen, Carlsbad, CA) was employed so that each sample could be used for both western blotting and mRNA quantification. Briefly, RNA was extracted from each cord sample individually using TRIzol® reagent and chloroform, then purified with the Qiagen RNeasy kit (#74104, Qiagen, Hilden, Germany). RNA concentration was measured using the Nanodrop system (Thermo Scientific, Wilmington, DE). Complementary DNA (cDNA) conversion was performed from 1 μg of RNA using a Veriti thermal cycler (Applied Biosciences, Waltham, MA) and a high-capacity cDNA conversion kit (Applied Biosciences, Waltham, MA) using the manufacturer’s protocol. Quantitative real-time PCR (qRT-PCR) was performed using the StepOnePlus Real-Time PCR System (Applied Biosciences, Waltham, MA) according to the manufacturer’s instructions. To determine changes in gene expression, primers were designed using the Primer-Blast tool, then obtained from Integrated DNA Technologies (IDT, Coralville, IA; see Table 2 for detailed primer information). Reported gene expression values were determined using the ΔΔ CT method: raw value of each sample normalized first to internal control (GAPDH) and then to the control group (3 months).

**Table 2:** Gene primers tested in comparative RT-PCR experiments

Gene	Sense	Antisense
CD86	5′-TGCTCATCTAAGCAAGGATACCCG-3′	5′-CGACTCGTCAACACCACTGTCCTG-3′
CD206	5′-GGATTGTGGAGCAGATGGAAG-3′	5′-CTTGAATGGAAATGCACAGAC-3′
p22^PHOX^	5′-GACGCTTCACGCAGTGGTACT-3′	5′-CACGACCTCATCTGTCACTGG-3′
p47^PHOX^	5′-CAGAATGTTGCCTGGTTG-3′	5′-GTCCCCTCCCTTAGATGA-3′
gp91^PHOX^	5′-CTTCACACGGCCATTCACAC-3′	5′-GTCATAGGAGGGTTTCCGGC-3′
TNFα	5′-GGCAGCCTTGTCCCTTGAAGAG-3′	5′-GTAGCCCACGTCGTAGCAAACC-3′
NFκB	5′-GTGCAGAAAGAAGACATTGAGGTG-3′	5′-AGGCTAGGGTCAGCGTATGG-3′
iNOS	5′-CAGCCCTCAGAGTACAACGAT-3′	5′-CAGCAGGCACACGCAATGAT-3′
Arg-1	5′-CAGTATTCACCCCGGCTACG-3′	5′-GCCTGGTTCTGTTCGGTTTG-3′
CD68	5′-TGTACCTGACCCAGGGTGGAA-3′	5′-GAATCCAAAGGTAAGCTGTCCGTAA-3′
IL10	5′-CAAGGAGCATTTGAATTCCC-3′	5′-GGCCTTGTAGACACCTTGGTC-3′
GAPDH	5′-GCTGGTCATCAACGGGAAA-3′	5′-ACGCCAGTAGACTCCACGACA-3′

Table provides sense and antisense sequences for each gene tested in this study. Genes investigated that showed no significant difference by age in either naïve or injured animals were omitted from results

### Western blot and Oxyblot

Protein from frozen tissue samples was isolated in TRIzol® reagent (Invitrogen, Carlsbad, CA). Detection of protein oxidation was performed by processing protein samples using the Oxyblot Protein Oxidation Detection Analysis Kit (Millipore, Billerica, MA) according to the manufacturer’s protocol. For both Oxyblot and western blotting, 15 μg of sample protein was run in a Mini-PROTEAN® TGX™ Precast Gel (Bio-Rad, Hercules, CA) and transferred to a Trans-Blot® Turbo™ (Bio-Rad) nitrocellulose membrane. Antibodies raised against 3-nitrotyrosine (5 μg/ml; Abcam, Cat# ab61392, RRID:AB_942087) were probed overnight at 4 °C. Immune complexes and oxidated proteins were detected with appropriate secondary antibodies and chemiluminescence reagents (Pierce).

For both Oxyblot and western blots, GAPDH (0.5 μg/ml; Millipore, Cat# MAB374 RRID:AB_2107445) was used as a control for gel loading and protein transfer. NIH ImageJ was used to assess pixel density of resultant blots for quantitation.

### Statistics

Sample sizes were calculated based on our previous work [34]. All assays were carried out by investigators blinded to subject group. Quantitative data are presented as mean ± standard error of the mean (SEM). General motor performance using the BBB test was analyzed using a two-way ANOVA with Tukey’s multiple comparison’s post hoc test. Ability to plantar step in footprint analysis task was analyzed using Chi-square analysis. Footprint analysis test, immunohistochemistry, biochemistry, and qRT PCR were analyzed using the Student *t* test. All statistical tests were performed using the GraphPad Prism Program, Version 6.03 for Windows (GraphPad Software, San Diego, CA). A *p* value < 0.05 was considered statistically significant.

## Results

### Uninjured middle-aged animals show altered stepping pattern in footprint analysis

**Fig. 1:**
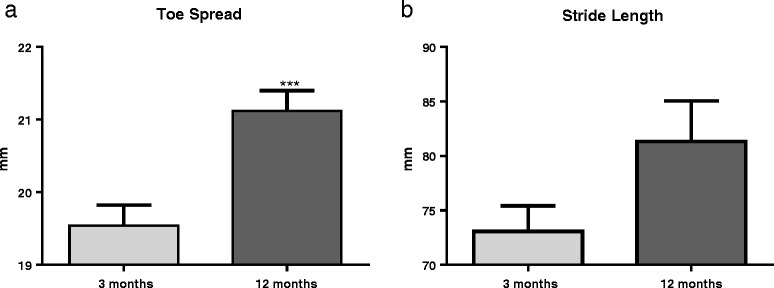
Uninjured aged animals show altered stepping pattern in footprint analysis. **a** Uninjured 12-month-old rats (*n* = 10) have significantly wider toe spread than 3-month-old rats (*n* = 12). **b** Uninjured 12-month old-rats show a trend toward increased stride length compared to 3-month-old rats, supporting toe spread findings. ***p < 0.001. *Bars* represent mean ± SEM

Footprint analysis of toe spread and stride length in uninjured animals revealed an alteration in motor function in middle-aged animals compared to young animals (Fig. 1a). Toe spread was measured from the thumb toe (toe 1) to the pinky toe (toe 5) in the hind paws. Twelve-month-aged rats showed a significantly wider toe spread than 3-month-aged rats (*t*(20) = 4.98, *p* = 0.0005). Stride length was measured as the length in millimeter between each consecutive step in the hind paws. Though not significant, 12-month-aged rats showed a trend toward longer strides than 3-month-aged rats (Fig. 1b; *t*(20) = 1.97, *p* = 0.0627), indicating that overall stepping patterns are altered with age.

### Microglial activation and pro-inflammatory gene expression is altered with age

**Fig. 2:**
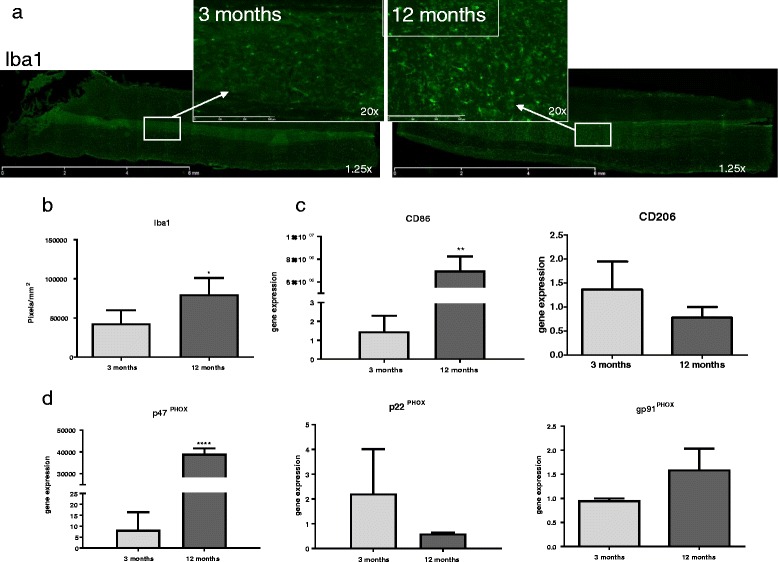
Microglia and NOX2 component activation and gene expression is altered with age. **a** Fluorescent microscope images of longitudinal sections of naïve 3- and 12-month-old rat spinal cords. Sections were stained with Iba-1 (*green*). Sections were imaged at 1.25× magnification and encompass an entire 10-mm section in the thoracic spinal cord. *Inserts* were taken at 20× magnification in the gray matter delineated by *white boxes* and *arrows*. **b** Immunostaining shows age-related differences in the microglial marker Iba1, showing significantly increased staining in 12- versus 3-month-old rats (size bar = 100 um). *N* = 4/group. **c** Comparative qPCR reveals significant increase in gene expression of the pro-inflammatory phenotype marker CD86. **d** Comparative RT-PCR reveals significant increase in p47^PHOX^ gene expression in 12-month-aged rats compared to 3-month-aged rats. No significant difference was seen in gene expression of p22^PHOX^ or gp91^PHOX^ between the two groups. *N* = 4/group. *p < 0.05, **p < 0.005, ****p < 0.0005. *Bars* represent mean ± SEM

To determine if aged tissue showed basal differences in microglial activation, uninjured tissue from 3- and 12-month-old rats was immunostained with the microglial marker Iba-1 (Fig. 2a) and processed for comparative RT-PCR for expression genes associated with microglial activation and NOX2 components. Our findings showed significantly increased Iba-1 staining (*t*(6) = 2.59, *p* = 0.0496) in 12-month-aged rat tissue compared to 3-month-aged rats (Fig. 2b). Comparative RT-PCR results (Fig. 2c) showed significantly increased gene expression in 12-month-aged rats of the pro-inflammatory phenotype marker CD86 (*t*(6) = 7.46, *p* = 0.0050) compared to 3-month-aged rats. No significant difference by age was found in any other genes probed, including the anti-inflammatory phenotype marker CD206, transcription factor NFκB, or inflammatory cytokine TNFα (Table 3). Relative fold changes between 3- and 12-month-old rats for all genes tested listed in numerical form can be found in Table 3 under “naïve.”

**Table 3:** Comparative gene fold changes for all genes tested by injury group

Comparative fold change by injury group				
Naïve		Gene tested	30 dpi	
3 months old	12 months old		3 months old	12 months old
1.467 ± 0.8292	**6.993 × 10^6^ ± 1.258 × 10^6^	CD86	1.096 ± 0.2886	***1.253 × 10^6^ ± 145,818
1.371 ± 0.5850	0.7830 ± 0.2167	CD206	1.104 ± 0.3465	**139,991 ± 24,361
2.217 ± 1.785	0.6052 ± 0.04079	p22^PHOX^	1.030 ± 0.1825	*20678 ± 6017
8.352 ± 8.037	****39,131 ± 2480	p47^PHOX^	7.739 ± 5.672	*1.225 × 10^6^ ± 473,918
0.9453 ± 0.05264	1.583 ± 0.4531	gp91^PHOX^	0.9421 ± 0.08263	*1.948 ± 0.4574
3.229 ± 2.839	163.1 ± 141.8	TNFα	0.6954 ± 0.3801	*11.55 ± 2.502
1.226 ± 0.5548	0.8387 ± 0.6209	NFκB	1.027 ± 0.1751	*4.108 ± 0.7734
44.06 ± 22.85	0.01669 ± 0.01419	iNOS	1.109 ± 0.3358	1.069 ± 0.4000
3.118 ± 2.773	1230 ± 794.0	Arg-1	1.047 ± 0.2304	1.382 ± 0.03944
0.4958 ± 0.4531	0.3324 ± 0.02472	CD68	1.104 ± 0.3346	1.011 ± 0.3394
2.438 ± 1.521	7.806 ± 4.643	IL10	1.051 ± 0.2427	1.057 ± 0.1975

Table demonstrates how age affects gene expression before and after injury. The average of the 3-month-old group in both the naïve and injury groups was considered the control mean value. Each individual sample’s fold change was compared to the control mean. Fold changes are presented in table as mean values of all samples in each group ± SEM. *N* = 4/group

*p < 0.05, **p < 0.005, ***p < 0.0005

### Middle-aged rats show increased gene expression of NOX2 cytosolic subunit component p47^PHOX^

To determine if NOX2 activity and expression were influenced by age, uninjured tissue from 3- and 12-month-old rats was processed for comparative RT-PCR and analyzed for target gene expression. Gene expression of the activated cytosolic subunit p47^PHOX^ was significantly increased in 12-month-aged rats compared to that in 3-month-aged rats (*t*(6) = 3.47, *p* < 0.0001; Fig. 2d). However, gene expression of the activated membrane bound NOX2 components p22^PHOX^ and gp91^PHOX^ did not reveal any significant difference by age.

### ROS production is altered with age

**Fig. 3:**
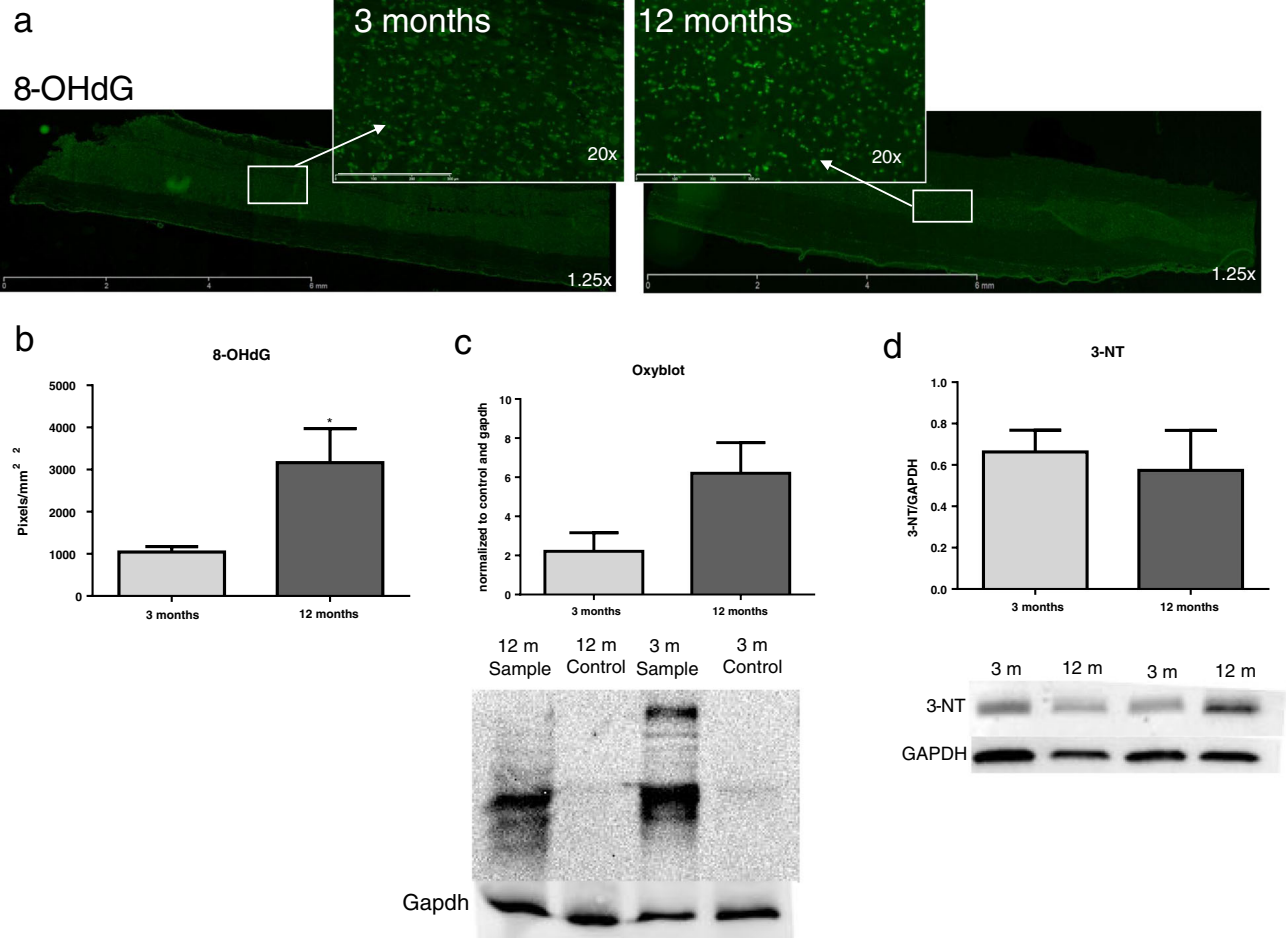
ROS production is increased with age. **a** Fluorescent microscope images of longitudinal sections of naïve 3- and 12-month-old rat spinal cords. Sections were stained with 8-OHdG (*green*). Sections were imaged at 1.25× magnification and encompass an entire 10-mm long section in the thoracic spinal cord. *Inserts* were taken at 20× magnification in the gray matter delineated by *white boxes* and *arrows*. **b** 8-OHdG stain shows significantly greater oxidative damage in 12 versus 3-month-old rats (size bar = 300 um). *N* = 4/group. **c** Oxyblot analysis shows a trend (*p* = 0.0946) toward increased protein oxidation in 12-month-aged rats compared to 3-months-aged rats. **d** Western blot analysis that shows protein expression of 3-NT, a marker of nitrosylated proteins which is a product of oxidative stress, is not significantly different between 12- and 3-month-aged rats. *N* = 4/group. *p < 0.05. *Bars* represent mean ± SEM

To determine if tissue showed basal differences in ROS production with age, uninjured tissue from 3- and 12-month-old rats was immunostained with the DNA oxidation marker 8-OHdG (Fig. 3a), Oxyblot analysis was performed to determine protein oxidation, and western blotting was performed to determine levels of 3-NT, a marker of protein nitrosylation. Our findings showed that 12-month-aged rats compared to 3-month-aged rats demonstrated a significant increase in 8-OHdG staining (*t*(6) = 3.06, *p* = 0.0281; Fig. 3b) and a trend toward increased protein oxidation via Oxyblot (*t*(6) = 2.18, *p* = 0.0946; Fig. 3c). Western blotting did not show any difference in protein nitrosylation between the two ages (Fig. 3d).

### Middle-aged rats show diminished motor function recovery after spinal cord injury

**Fig. 4:**
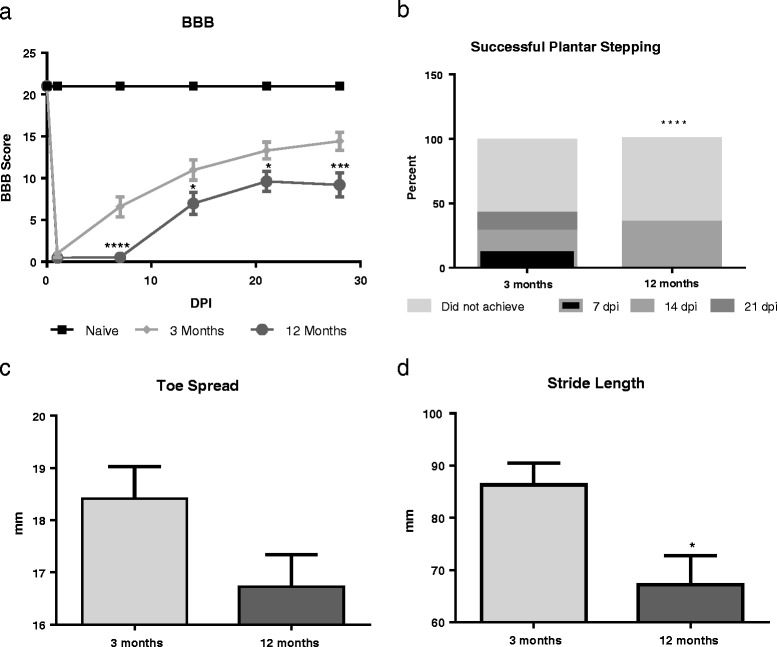
Aged animals show diminished motor function recovery after injury. **a** At 1 dpi, no difference in performance in BBB scores were observed in injured rats. At all other time points (7, 14, 21, and 28 dpi), 12-month-old injured rats (*n* = 17) showed significantly reduced BBB scores compared to 3-month-old rats (*n* = 18), indicating significantly impaired recovery to injury. **b** Aged animals show impaired ability to plantar step and complete footprint analysis task. Twelve-month-old rats (*n* = 17) show significantly diminished ability to plantar step in order to complete the footprint analysis task compared to 3-month-old rats (*n* = 14), indicating a diminished ability to take steps or strides. **c** At 28 dpi, toe spread analysis showed a trend in 12-month-old rats (*n* = 7) toward narrower toe spread compared to 3-month-old rats (*n* = 5). **d** 12-month-old rats (*n* = 7) showed significantly decreased stride length compared to 3-month-old rats (*n* = 5). *p < 0.05, **p < 0.005, ***p < 0.0005, ****p < 0.0001. *Bars* represent mean ± SEM

Next, we examined both middle-aged and young animals after moderate contusion SCI to investigate how age alters response to injury. Our results from the BBB general motor test showed worsened performance by aged animals after injury (Fig. 4). Twelve-month-old rats showed worsened performance compared to 3-month-old rats at all time points except 1 dpi, indicating significantly impaired recovery to injury [F(1.33) = 12.81, *p* < 0.0001; Fig. 4a]. At 1 dpi, 12- and 3-month-old rats averaged a score of 0.471 ± 0.2827 and 1.000 ± 0.485 respectively, indicating no stepping ability with only slight movement of one joint on one limb. At 7 dpi, the 3-month-old group has progressed to a mean of 6.583 ± 1.188, indicating extensive movement in all multiple joints, while the 12-month-old group remained at an average score of 0.529 ± 0.208. By 14 dpi, the 3-month-old group continued to show steady improvement with a BBB score of 10.970 ± 1.217, with occasional weight-supported steps but no limb coordination, and the 12-month-old group showed sweeping of the feet with no weight-bearing steps, averaging 6.971 ± 1.309. At 21 and 28 dpi, improvement in the 3-month-old group was still steady (13.310 ± 1.009 and 14.42 ± 1.070, respectively), showing consistent weight-bearing plantar stepping with coordinated limb movements, while the 12-month-old group reached a max performance of 9.618 ± 1.202 at 21 dpi, with weight-bearing plantar stepping but only occasional coordinated limb movements and never improved beyond that score. This comparison demonstrates not only that the 12-month-old groups’ recovery was diminished in comparison with that of the 3-month-old but also that the overall speed of recovery was delayed—the improvements in functional performance in the 12-month-old group occurred at least 1 week after the same score had been achieved in the 3-month-old group.

The number of rats that achieved plantar stepping, enabling them to complete footprint analysis, at each time point through 28 days was assessed and found to differ by age. The number of 12-month-aged rats that achieved plantar stepping by 28 dpi was significantly lower than the 3-month-aged rats [c^2^(3, *N* = 31) = 37.52, *p* < 0.0001; Fig. 4b]. Rats that recovered the ability to take plantar steps with their hind limbs were tested on the footprint analysis task. At 28 dpi, 12-month-old rats that were able to plantar step demonstrated a significantly shorter stride length (*t*(10) = 2.53, *p* = 0.0297; Fig. 4d) and a trend toward narrower toe spread (*t*(10) = 1.192, *p* = 0.0967; Fig. 4c) in comparison to the 3-month-old rats.

### Middle-aged rats have significantly greater lesion volume rostral and caudal to the lesion epicenter

**Fig. 5:**
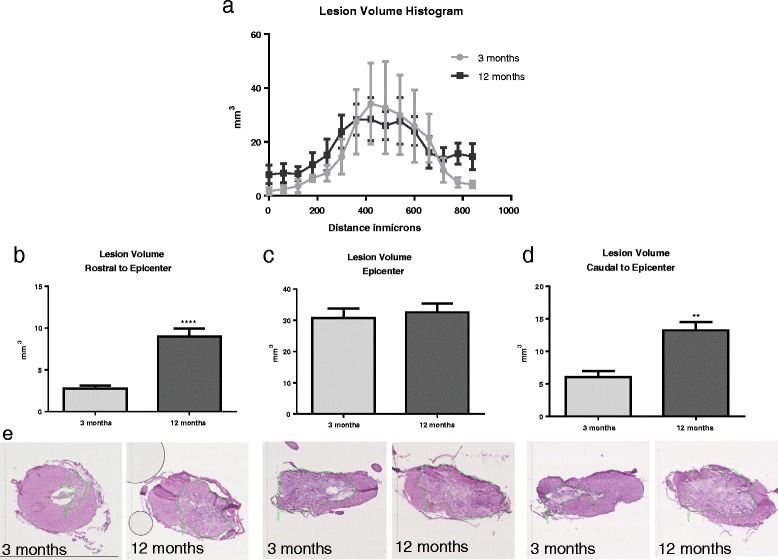
Aged rats have significantly great lesion volume rostral and caudal to the lesion. **a** Quantitative analysis of tissue stained with H&E reveals that at 30 dpi, 12-month-old rats show an increased lesion volume compared to 3-month-old rats both rostral and caudal to the injury epicenter, suggesting that aged rats have an increased volume and length of lesion compared to young. Lesion volume histogram shows the pattern of lesion spread from the epicenter. **b** Lesion volume rostral to the epicenter (0–180 μm) was significantly greater in 12-month-old rats compared to 3-month-old rats. **c** No significant difference was found in lesion volume at injury epicenter (360–540 μm). **d** Lesion volume caudal to the epicenter (720–900 μm) was significantly greater in 12-month-old aged rats compared to 3-month-old rats. **e** Photos depict sections of the spinal cord at each segment through the lesion from both 3- and 12-month-old rats. Rostral image is from a 120-μm section, epicenter image is from a 400-μm section, and caudal image is from a 820-μm section. Size bar = 3 mm. *N* = 6/group. **p < 0.005, ****p < 0.0001. *Bars* represent mean ± SEM

At 30 dpi, results from our lesion volume analysis showed a larger lesion volume in 12-month-old rats compared to that in 3-month-old rats; however, the volume of the lesion varied by proximity to lesion epicenter (Fig. 5a). At the lesion epicenter (360–540 μm), both 3- and 12-month-old animals demonstrated marked tissue damage and loss, with no significant difference in volume between age groups (Fig. 5c). But in both rostral (0–180 μm) and caudal (720–900 μm) lesion epicenters, 12-month-old animals had significantly greater lesion volumes than 3-month-old animals (*t*(10) = 6.09, *p* < 0.0001/rostral, and *t*(10) = 4.64, *p* = 0.0017/caudal; Fig. 5b and d).

### Microglial activation and gene expression 30 days after spinal cord injury is altered with age

**Fig. 6:**
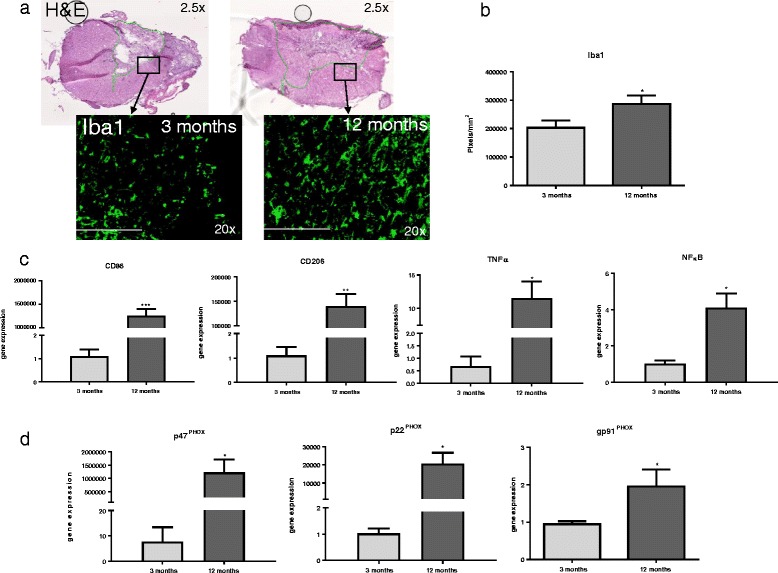
Microglial and NOX2 component activation and gene expression after spinal cord injury at 30 dpi. **a** 30 dpi H&E-stained images of 3 and 12-month-old injured spinal cord. *Black boxes* depict location of Iba1-stained quantification, in the closest intact tissue outside the central cavity of the injury. *Black arrows* point to corresponding images of immunofluorescent staining of Iba1. **b** Twelve-month-aged rats showed significantly greater staining in comparison to 3-month-aged rats at 30 dpi. *N* = 4/group. **c** Comparative RT-PCR reveals significant increase in gene expression of the M1 phenotype marker cd86, M2 phenotype marker cd206, and inflammatory cytokines TNFα and NFκB in 12-month-aged rats compared to that in 3-month-aged rats, suggesting an overall increase in microglial gene expression after injury with age. **d** Comparative RT-PCR of NOX2 components p47^PHOX^, p22^PHOX^, and gp91^PHOX^ reveal a significant increase in gene expression of all three genes in 12-month-aged rats compared to 3-month-aged rats at 30 dpi. *N* = 4/group. *p < 0.05, **p < 0.005, ***p < 0.0005. *Bars* represent mean ± SEM

Immunostaining at 30 dpi in both 3- and 12-month-aged tissues revealed increased microglial activation, as defined by cell size and number, and reflected in pixel density of Iba1 staining microglia in comparison to naïve tissue (Fig. 6a). Aged tissue showed significantly greater Iba1 staining compared to young tissue (*t*(6) = 2.16, *p* = 0.0487; Fig. 6b). Comparative RT-PCR results (Fig. 6c) showed significantly increased gene expression in 12-month-aged rat compared to 3-month-aged rats of pro-inflammatory marker CD86 (*t*(6) = 7.26, *p* = 0.0050), anti-inflammatory marker CD206 (*t*(6) = 5.75, *p* = 0.0045), inflammatory cytokine TNFα (*t*(6) = 3.47, *p* = 0.0127), and transcription factor NFκB (*t*(6) = 4.98, *p* = 0.0156). Relative fold changes between 3- and 12-month-old rats for all genes tested listed in numerical form can be found in Table 3 under “30 dpi”. An additional experiment comparing gene expression between naïve and injured groups within each age group was performed on selected genes to verify gene expression patterns (Table 4).

**Table 4:** Comparative gene fold changes by age group for significantly impacted genes

Comparative fold change by age group				
3 months old		Gene tested	12 months old	
Naïve	30 dpi		Naïve	30 dpi
1.467 ± 0.8292	4.481 ± 1.179	CD86	0.6781 ± 0.3546	0.7444 ± 0.08664
1.731 ± 0.5850	0.7586 ± 0.5561	CD206	1.094 ± 0.3027	111,858 ± 67,300
1.043 ± 0.2966	225.2 ± 76.85	p22^PHOX^	1.005 ± 0.06771	218.9 ± 132.1
1.491 ± 0.8159	3.134 ± 2.551	p47^PHOX^	1.232 ± 0.4360	*180,416 ± 31,468
1.001 ± 0.02864	28.02 ± 3.662	gp91^PHOX^	1.154 ± 0.3588	*54.89 ± 21.85

Table demonstrates the effect of injury on gene expression within age groups. The average of the naïve in both the 3- and 12-month-age groups was considered the control mean value. Each individual sample’s fold change was compared to the control mean. Fold changes are presented in the table as mean values of all samples in each group ± SEM. *N* = 4/group

*p < 0.005

### Middle-aged rats show increased NOX2 component gene expression at 30 dpi

To determine if NOX2 activity and expression after injury were influenced by age, tissue from injured 12- and 3-month-aged rats at 30 dpi was processed for comparative RT-PCR and analyzed for expression of target genes. Gene expression of the cytosolic subunit p47^PHOX^ and the membrane components p22^PHOX^ and gp91^PHOX^ were significantly increased in 12-month-aged rats compared to 3-month-aged rats (*t*(6) = 3.47, *p* = 0.0404; *t*(6) = 4.61, *p* = 0.0182 and *t*(6) = 2.94; *p* = 0.0186, respectively; Fig. 6d).

### Middle-aged rats show increased ROS production and oxidative stress at 30 dpi

**Fig. 7:**
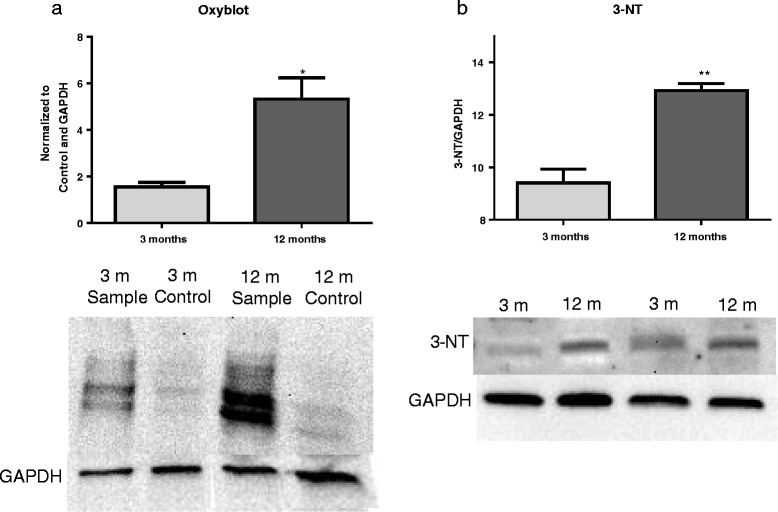
Aged rats show increased ROS production and oxidative stress at 30 dpi. **a** Oxyblot analysis reveals a significant increase in protein oxidation in 12-month-aged rats compared to 3-month-aged rats at 30 dpi. **b** Western blot analysis reveals a significant increase in 3-NT, a marker of nitrosylated proteins which is a product of oxidative stress, in 12-month-aged rats compared to that in 3-month-aged rats 30 dpi. *N* = 4/group. *p < 0.05, **p < 0.005. *Bars* represent mean ± SEM

To determine if injured aged tissue showed differences in ROS production, tissue from 3- and 12-month-old rats was processed for Oxyblot and western blotting at 30 dpi. Oxyblot analysis revealed that 12-month-aged rats when compared to 3-month-aged rats demonstrated significantly increased protein oxidation (*t*(6) = 4.04, *p* = 0.0156; Fig. 7a), and western blotting of 3-NT, a marker of protein nitrosylation, demonstrated significantly increased protein nitrosylation (*t*(6) = 1.758, *p* = 0.0038; Fig. 7b).

## Discussion

This study demonstrates that aging alters the inflammatory environment in the 12-month-old rat spinal cord. Uninjured 12-month-old, middle-aged rats show increased microglial activation and NOX2 cytosolic subunit and pro-inflammatory gene expression and increased DNA oxidation when compared to 3-month-old, young adult rats. At 30 dpi, middle-aged rats show decreased functional recovery and increased lesion volume, microglial activation, NOX2 component and inflammatory gene expression, and protein oxidation and nitrosylation compared to young adult rats.

Previous work has shown increasing oxidative stress with age [8]. We now demonstrate that this increase is obvious in the CNS, with increased markers of oxidative stress in the middle-aged rat spinal cord both before and after injury. In naïve middle-aged rats compared to young adult, increases in the DNA peroxidation marker 8-OHdG, but not of protein carbonylation (Oxyblot) and nitrosylation (3-NT), were observed. As mutations in DNA have been found by early middle age [5], DNA peroxidation may be one of the first signs of increasing ROS production, as part of a pro-inflammatory environment in an aging tissue [14]. As protein modifications occur further downstream than DNA, age-related increases in protein carbonylation and nitrosylation markers may be more indicative of a hyperactive inflammatory response to injury.

The increased ROS production found in middle-aged rats coincides with our results showing increased microglial activity with age. Increased staining of the microglial marker Iba1 was coupled with increased pro-inflammatory gene expression of CD86 in middle-aged rats, suggesting that microglia are present in aged tissue in a pro-inflammatory state. Further, staining demonstrates alterations in microglial phenotype as defined previously [11], with 3-month-old rats showing a non-activated phenotype with lengthy processes and a small cell body and 12-month-old rats showing a partially activated microglial phenotype, with an engorged, rounder shaped cell body. These findings in the spinal cord are similar to previous findings in the brain [14], suggesting an age-related pattern of the inflammatory response that is not impacted by regional differences between the spinal cord and the brain [17].

Interestingly, our gene expression experiments at 30 dpi showed an increase in both pro-inflammatory- and anti-inflammatory-related genes, suggesting that chronically after injury, all microglial activities are elevated, not only pro-inflammatory as seen in uninjured tissue. Previous work using aged mouse and female rat models of traumatic injury has shown a decrease in anti-inflammatory signaling and an increase in pro-inflammatory signaling at 24 h and 7 dpi [18, 19, 21, 25]. In a traumatic brain injury model with 23-month-old mice, a similar increase in both pro- and anti-inflammatory gene expression was observed at 24-h post-injury [35].Our findings at 30 dpi suggest that a change in activation type may occur chronically post-injury that leads to a more active overall inflammatory response. Further, Morganti et al. [35] found that acute gene expression alterations were strongly dependent on peripheral monocyte infiltration; it is possible that the 30 dpi data in our study is more microglial related due to the reduction in macrophage invasion at this later time point [36]. Future work should evaluate these earlier time points and contribution of peripheral and central inflammatory cells in 12-month-old rats to fully characterize our rodent model.

Increased gene expression of the activated NOX2 cytosolic subunit p47^PHOX^ but not the membrane components p22^PHOX^ or gp91^PHOX^ suggests that NOX2 expression is increased in middle-aged naïve rats, corresponding with the observed pro-inflammatory microglia. At 30 dpi, gene expression of both NOX2 membrane components p22^PHOX^ and gp91^PHOX^ and the cytosolic subunit p47^PHOX^ are elevated in middle-aged rats compared to young adults, suggesting that microglial NOX2 is “primed” prior to injury and activated more fully with injury. This could play a role in the increased ROS production we see in a middle-aged spinal cord. We and the others have shown that NOX2 is involved in microglial ROS production [22–24].

Our lesion volume experiments at 30 dpi showed that at the epicenter of the injury, the lesion volume was not significantly different by age, but was significantly increased in middle-aged rats compared to young adults in sections of the spinal cord both rostral and caudal to the injury, demonstrating an increased volume farther from the lesion epicenter in middle-aged rats. Our results coincide with previous work showing increased lesion volume in aged female rats after SCI [19] and in a mouse model of TBI [25]. These findings demonstrate that injury severity is increased in middle-aged rats, which may be a result of the exaggerated inflammatory response leading to increased oxidative stress on cells. Control of motor function is distributed throughout the spinal cord with multiple interacting tracts, and recovery of function observable in injury models is largely due to the ability of the spinal networks to compensate for some loss of tissue [37]. Interestingly, previous work has shown that the increase of lesion volume and functional deficits is not completely linear, but with increasing injury severity, once a threshold proportion of tract is lost, loss of function is seen [38, 39]. The increased lesion volume found in our middle-aged rats would impact a greater number of tracts in the spinal cord, likely surpassing that threshold and contributing to the delayed functional recovery we observed.

Interestingly, naïve middle-aged rats also show functional impairments, with altered stepping patterns with wider toe spread and longer stride length compared to young adult rats. Functional performance was more prominently affected in middle-aged rats than young adult after contusion SCI, at all time points examined out to 30 dpi. Middle-aged rats compared to young adult rats showed age-related delays in motor function with delayed general functional recovery, as demonstrated by lower scores on the BBB test, and worsened ability to complete the footprint task. These results agree with previous work in mouse and male and female rat models of aged SCI [19, 21, 40]. These previous studies demonstrate that 18-month-old female rats showed elevated base of support during walk both before and after injury [19], similar to our studies, while 12-month-old male rats consistently demonstrate impaired BBB function by 7 dpi [40]. In contrast, 14-month-old male mice did not demonstrate functional impairment before injury, although this may be due to the use of only the locomotor score, similar to the BBB; however, a significant reduction in locomotor score was noted after injury [21], as in our study.

Interestingly, footprint analysis in middle-aged rats after SCI are opposite of what was seen in naïve, showing decreased stride lengths and narrower toe spread at 30 dpi versus increased stride length and wider toe spread in naïve. Previous work in mouse models of SCI have shown that both stride length and toe spread decrease significantly after injury [41–43] and have suggested that the decrease may be indicative of sensory motor function deficits in the hind limbs due to injury. Thus, our findings of impaired recovery and altered stepping pattern in footprint analysis may be associated with an age-related decrease in precise motor control in the hind limbs.

## Conclusions

Overall, the results of our study suggest that increasing age leads to a pro-inflammatory environment, with alterations to microglial activation, NOX2 enzyme component expression, and an increase in oxidative stress in the cellular environment that may contribute to worsened outcomes after SCI. The initiating factor in these effects and the cause/effect relationship between each remains unclear and is an important consideration for the field moving forward. It is clear that there are intimate relationships between inflammation, oxidative damage, and NOX2, and the current work contributes to understanding that all three play a role in motor function and post-injury recovery. However, more work is needed to understand which, if not all three, the essential component is. We currently hypothesize that age-related changes to ROS production caused by increased microglial activation and NOX2 expression are associated with an exaggerated chronic inflammatory response to injury.

With the rise in SCI in the aging population, the results of this study suggest that increased inflammatory response may be an important factor to be considered in therapeutic interventions after injury and that age should be considered when developing a therapeutic treatment plan. Further, previous work has demonstrated that inhibition of the NOX enzyme can decrease oxidative stress and inflammation. Due to the association of NOX2 with both ROS production and microglial activity, our findings suggest a potential avenue for NOX2 inhibitory treatment options to quell the exaggerated inflammatory response and should be a topic of interest for future research.

## Abbreviations

CNS: Central nervous system
DPI: Days post-injury
NOX: NADPH oxidase
ROS: Reactive oxygen species
SCI: Spinal cord injury
